# Research progress of interleukin-15 in cancer immunotherapy

**DOI:** 10.3389/fphar.2023.1184703

**Published:** 2023-05-12

**Authors:** Menghan Cai, Xuan Huang, Xiting Huang, Dianwen Ju, Yi Zhun Zhu, Li Ye

**Affiliations:** ^1^ School of Pharmacy and State Key Laboratory of Quality Research in Chinese Medicine, Macau University of Science and Technology, Macau, Macau SAR, China; ^2^ Minhang Hospital and Department of Biological Medicines at School of Pharmacy, Fudan University, Shanghai, China; ^3^ Shanghai Engineering Research Center of Immunotherapeutics, School of Pharmacy, Fudan University, Shanghai, China

**Keywords:** interleukin-15 agonist, cytokines, cancer, cancer immunotherapy, recombinant fusion proteins

## Abstract

Interleukin-15 (IL-15) is a cytokine that belongs to the interleukin-2 (IL-2) family and is essential for the development, proliferation, and activation of immune cells, including natural killer (NK) cells, T cells and B cells. Recent studies have revealed that interleukin-15 also plays a critical role in cancer immunotherapy. Interleukin-15 agonist molecules have shown that interleukin-15 agonists are effective in inhibiting tumor growth and preventing metastasis, and some are undergoing clinical trials. In this review, we will summarize the recent progress in interleukin-15 research over the past 5 years, highlighting its potential applications in cancer immunotherapy and the progress of interleukin-15 agonist development.

## 1 Introduction

IL-15, a stimulatory cytokine, has garnered considerable interest in recent years as a potential cancer immunotherapy drug, and was rated as the most promising candidate by the National Cancer Institute in 2008(Cheever, 2008). The use of immunotherapy as a cancer treatment strategy has revolutionized oncology. Immunotherapy works by activating the immune system to recognize and attack cancer cells, thus offering a novel approach to cancer treatment that is distinct from traditional chemotherapy and radiation. As our understanding of the immune system and its interactions with cancer cells has improved, researchers have identified many potential targets and endogenous biomolecules with anti-tumor effects. This has led to the development of a variety of cancer immunotherapeutic agents, such as recombinant stimulatory cytokines (e.g., IL-2, IFN-α), immune checkpoint inhibitors (e.g., anti-PD-1/PD-L1), and CAR-T cell therapy (Cortés-Selva et al., 2021; Kraehenbuehl et al., 2022; Propper and Balkwill, 2022). These agents have shown impressive results in treating many cancer types. However, despite these advances, cancer immunotherapy still faces challenges, such as identifying the most appropriate targets and predicting which patients will respond to treatment. One of the most promising targets for cancer immunotherapy is IL-15. IL-15 agonists have demonstrated significant inhibition of tumor growth and anti-metastatic properties in preclinical studies and are currently undergoing clinical investigation (Guo et al., 2017; Fiore et al., 2020; Bergamaschi et al., 2021). This article delves into the mechanism of action, immune function, and research progress of IL-15 in cancer immunotherapy, along with a detailed overview of the current development status of IL-15 agonists.

## 2 Biology of IL-15 and its receptor

### 2.1 Biology of IL-15

IL-15 is a cytokine, which means it is a signaling protein with a broad range of functions in the immune system. IL-15 is a 14–15 kDa glycoprotein encoded on human chromosome 4q31 (Anderson et al., 1995a; Anderson et al., 1995b) and is mainly secreted by dendritic cells (DC), macrophages, and monocytes (Doherty et al., 1996). It was isolated from the culture supernatant of monkey kidney epithelial cell line CV-1/EBNA by Grabstein et al. (1994), and was called “T cell growth factor”. IL -15 has a 4-α-helix bundle structure and belongs to the common cytokine receptor common gamma chain (*γ*_c_) family of cytokines along with IL-2, IL-3, IL-4, IL-6, and IL-21 (Grabstein et al., 1994; Yang and Lundqvist, 2020). They all play a role forming various immune cell populations, regulate cell differentiation, and, depending on the cellular context, either promote survival or induce apoptosis (Leonard et al., 2019).

Among them, IL-2 is the most most comprehensively one. It became the first immunotherapy approved by the United States Food and Drug Administration (FDA) for cancer treatment nearly 30 years ago (Yang and Lundqvist, 2020). Possessing common receptor subunits (shared *ß* and *γ* chains) with IL-12, IL-15 not only activates similar signaling pathways, but also possesses similar biological activities, including stimulating the proliferation and activation of T cells and NK cells, inducing the synthesis of B cell immunoglobulins and supporting the differentiation of cytolytic effector cells. However, IL-2 has the effect of promoting the proliferation of regulatory T cells and CD4^+^ helper T cells (Waldmann, 2015). It can also eliminate self-relative T cells via activation-induced cell death (AICD) to prevent autoimmunity (Lenardo, 1996; Waldmann, 2006). IL-15 not only supports the maintenance of long-lived memory phenotype CD8^+^ T cells and NK cells, but also inhabits AICD induced by IL-2 (Marks-Konczalik et al., 2000; Waldmann, 2018). In addition, due to the serious side effects and toxicity of IL-2 therapy, there are still many limitations in its clinical application. Therefore, IL-15 has gradually emerged as an alternative to IL-2 in cancer treatment by reason of its functional similarity to IL-2 with several added benefits (the difference between IL-12 and IL-15 is shown in Table 1) (Yang and Lundqvist, 2020). The physiological/pathological roles and mechanisms of action of IL-15 in immunity have been more thoroughly investigated. IL-15 is essential in the growth, mobilization and activation of immune cells such as natural killer (NK) cells, CD8^+^ T cells, γδ T cells and natural killer T (NKT) cells. IL-15 can also enhance the anti-tumor activity of immune cells (Zhang et al., 2021).

**TABLE 1:** Comparison of interleukin-2 and interleukin-15.

Properties	IL-2	IL-15
Structure	15.5 kDa; 133 amino acids; four-helix bundle	14–15 kDa; 114 amino acids; four-helix bundle
Main site of synthesis	Four exons; chromosome 4q26	Eight exons; chromosome 4q31
Receptor	Cis-presentation to IL2Rα, IL2/IL15Rβ, and γc coexpressed on activated T and B cells	IL-15Rα on the surface of monocytes and dendritic cells trans-presents IL-15 to NK cells and CD8^+^ memory T cells expressing IL-2/15Rβ and γc
Gene structure and location	Activated T cells	Dendritic cells and monocytes
Common function	Proliferation and differentiation of NK cells, and T and B cells	
Unique function	Maintenance of Tregs and elimination of self-reactive T cells mediated by AICD to yield self-tolerance	Maintenance of NK cells and CD8^+^CD44^hi^ memory T cells to provide a long-term immune response to pathogens
Major challenges and adverse effects	Short half-life; vascular leak syndrome and cardiotoxicity	Short half-life
Possible solutions to challenges	PEGylated agonists selective for low-affinity receptor IL-2Rβγ (such as bempegaldesleukin and nemvaleukin)	Superagonist comprising IL-15 bound to high-affinity receptor IL-15Rα (such as N-803)

Note: Data are from refs (Damjanovich et al., 1997; Waldmann, 2006; Waldmann, 2015; Propper and Balkwill, 2022).

In addition to its role in the immune system, IL-15 also has many other functions, such as protecting epithelial cells, keratinocytes, hepatocytes and fibroblasts from apoptosis, promoting angiogenesis with endothelial cells, and supporting the endurance of neurons (Budagian et al., 2006). Il-15 also has the potential to enhance lipid and glucose metabolism, reduce inflammation in white adipose tissue and improve mitochondrial function, suggesting a potential role in the treatment of obesity and type 2 diabetes (Ye, 2015; Duan et al., 2017). Additionally, Liu et al. (2022) revealed that ovarian granulosa cells were prevented from proliferating at high doses of IL-15. They believe that IL-15 plays a role in PCOS follicular atresia by encouraging the apoptosis of granulosa cells. It has also been shown that IL-15 not only promotes endurance and fuel supply, but also alleviate endoplasmic reticulum stress and improves cell survival, thus these findings support a role for IL-15 in the induction of exercise endurance and molecular adaptation in skeletal muscle (Quinn et al., 2013), as well as having the potential to treat cerebral ischemia (Nguyen et al., 2021; Zhu et al., 2022).

### 2.2 IL-15 receptors

Interleukin-15 (IL-15) is a cytokine that plays a critical role in the immune system’s function. The receptors for IL-15 are a heterotrimeric containing three subunits, which are IL-15 receptor alpha (IL-15Rα), IL-2/IL-15 receptor beta (IL-2/IL-15Rβ, also known as CD122), and the common gamma chain (*γ*_c_, also known as IL-2Rγ or CD132; Giri et al., 1995).The majority of lymphohematopoietic cells, such as T cells, NK cells, monocytes, and neutrophils, express IL-2/15R and *γ*_c_ in a restricted manner (Liao et al., 2013). IL-2Rβ and *γ*_c_ generally combine to form an IL-2Rβ/*γ*_c_ receptor complex, which is expressed on the surface of effector cells such as T cells and NK cells. As a signal receptor with moderate affinity to IL-15 (K_d_ ∼10^–9^ M), IL-2Rβ/*γ*_c_ is responsible for signaling upon binding to IL-15 (Fiore et al., 2020; Bernstein and Spangler, 2021).IL-15Rα is a type of cytokine receptor that is specific for IL-15. It binds with IL-15 with a high affinity by itself ((K_d_ ∼10^–11^ M)) and might be involved in certain intracellular signals (Ring et al., 2012).The sushi domain of IL-15Rα mediates its binding to IL-15 through ionic interactions (Lorenzen et al., 2006), which leads to a stable complex that is involved in the presentation of IL-15 (Dubois et al., 2002).Additionally, IL-15Rα may also be present in soluble form against excessive IL-15 activity (Dubois et al., 2002). IL-15Rα can also form high-affinity trimeric receptors with IL-2Rβ and *γ*_c_ chains, allowing the cells to respond even at low concentrations of IL-15 (Anderson et al., 1995a; Anderson et al., 1995b).In summary, IL-15Rα acts more like a stabilizing and synergistic element (chaperone molecule) of IL-15, assisting IL-15 in its trans-presentation (Fiore et al., 2020; Bergamaschi et al., 2021; Bernstein and Spangler, 2021).

## 3 Mechanisms of action of IL-15

### 3.1 Trans-presentation

**FIGURE 1:**
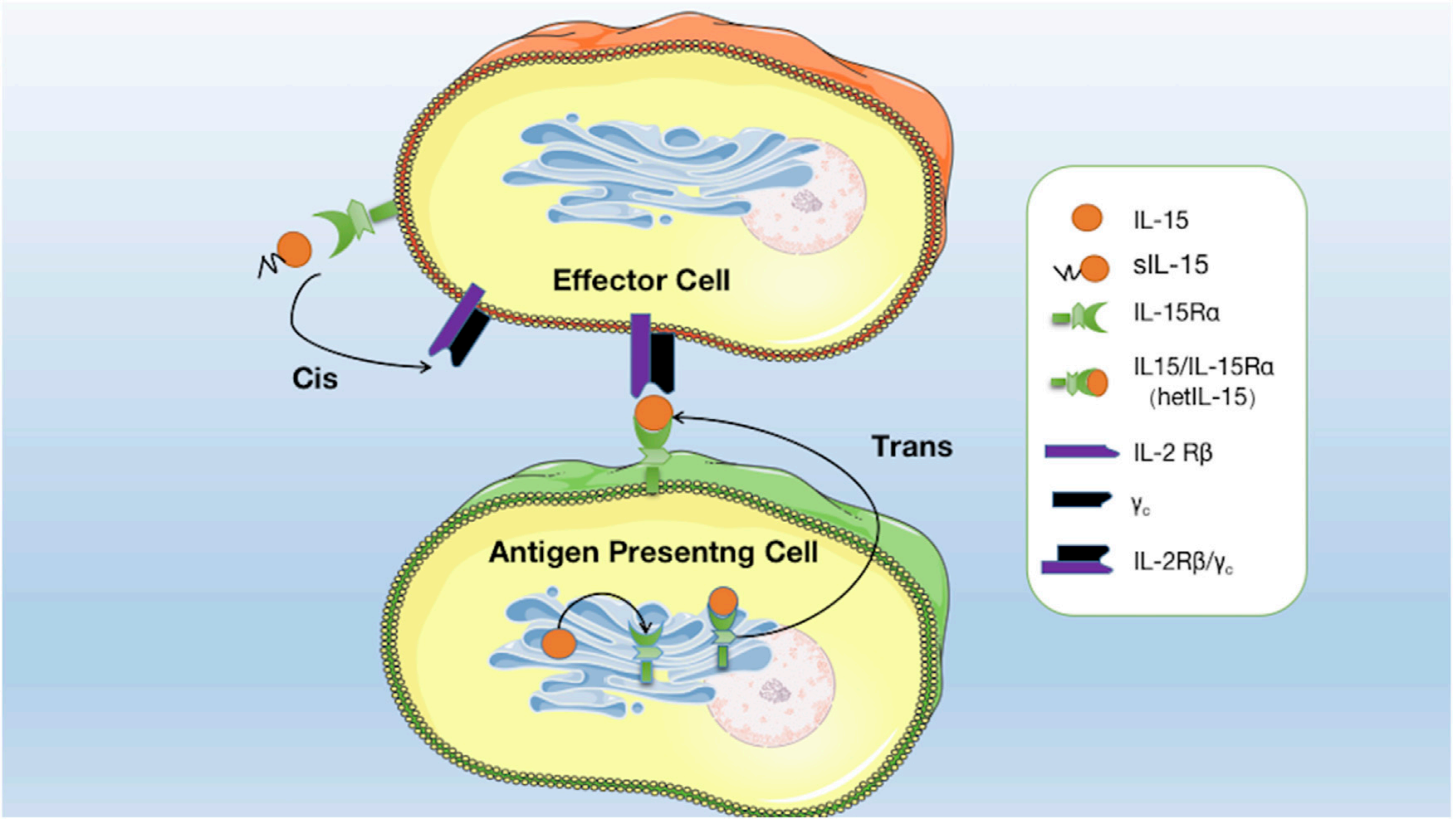
Mode of interaction of IL15 with its receptors: soluble IL-15 (sIL-15) binds directly to IL-15Rα on effector cells and is then presented to IL-2Rβ/*γ*_c_ receptors on the same effector cells. IL-15 binds to IL-15Rα in the endoplasmic reticulum to form hetIL-15, which is anchored on the surface of the APC to form an immune synapse and is then transmitted to adjacent effector cells and binds to IL-2Rβ/*γ*_c_ receptors to activate the signalling pathway.

IL-15 is a pleiotropic cytokine that plays a crucial role in developing and maintaining the immune system. One of the distinctive features of IL-15 is its capacity to undergo trans-presentation, a mechanism of cytokine signaling. Waldmann (2015) found the existence of co-expression of IL-15Rα with IL-15. They are mainly expressed in monocytes, macrophages, and dendritic cells by the stimuli such as type I/II interferon, CD40 signal, and lipopolysaccharide. IL-15Rα with IL-15 assemble in the endoplasmic reticulum to form an IL-15/IL-15Rα heterodimeric complex (hetIL-15), which is subsequently anchored on the surface of the antigen-presenting cell (APC) (Fiore et al., 2020). Contrary to the secretory mode of action, membrane-anchored hetIL-15 is mainly transmitted to adjacent IL-2Rβ/*γ*_c_^+^ effector cells by forming immune synapses, binding to IL-2Rβ/*γ*_c_ receptors on effector cells, and activating signaling pathways. This process is called trans-presentation (Fiore et al., 2020). Research has indicated that *in vivo*, trans-presentation is the primary mechanism by which IL-15 operates. This mode of action enables IL-15 to effectively exert biological activity on cells, even with minimal secretion (Figure 1) (Fiore et al., 2020).

### 3.2 Cis-presentation

In addition to monocytes, macrophages, and dendritic cells, IL-15Rα is expressed in effector cells such as NK cells, T cells, NKT cells, and B cells (Mortier et al., 2009; Fujii et al., 2018). Soluble IL-15 (sIL-15), partially secreted extracellularly, can activate signaling pathways by preferentially binding to IL-15Rα on effector cells before being presented to IL-2Rβ/*γ*_c_ receptors on the same cells. This process is known as cis-presentation, which is the secondary mode of action of IL-15 (Figure 1; Fiore et al., 2020). This mechanism allows for the local delivery of IL-15 signals to immune cells close to the APCs. IL-15 cis-presentation is distinct from the classic presentation of cytokines, in which cytokines are released by cells and bind to their receptors on distant target cells.

## 4 Immunostimulatory effects and anti-tumor potential of IL-15

**FIGURE 2:**
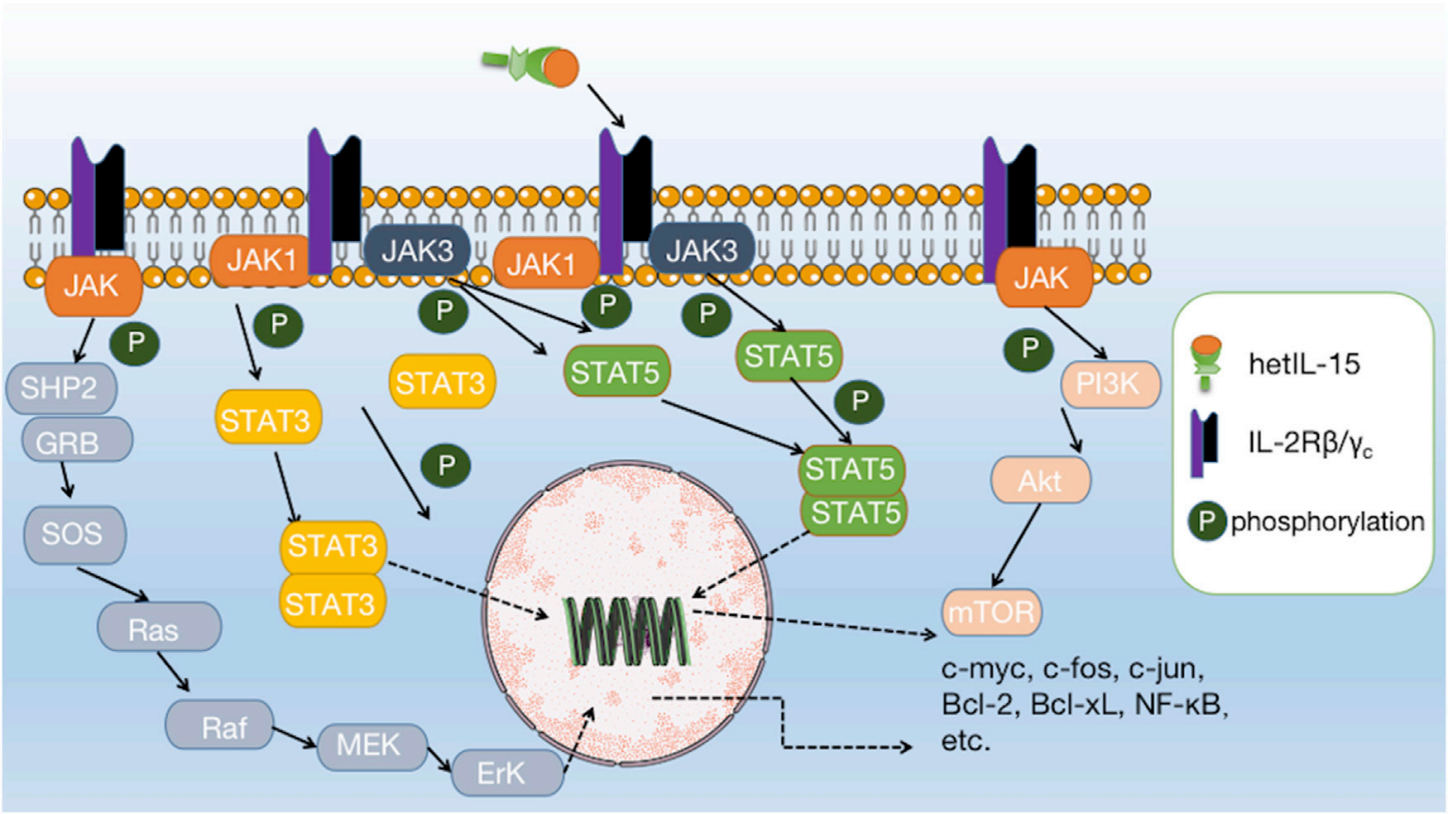
Interaction of IL-15 with its receptor and downstream signalling pathways. IL-15 binds to IL-2Rβ/*γ*_c_ and directly activates the JAK1/STAT3 and JAK3/STAT5 pathways, further initiating downstream pathways such as PI3K/AKT/mTOR, Ras/Raf/MAPK, and AKT-XBP1s.

IL-15 has been shown to possess significant anti-tumor potential, making it an attractive target for cancer immunotherapy. IL-15 binds to IL-2Rβ/*γ*_c_ and directly activates the JAK1/STAT3 and JAK3/STAT5 pathways at the intracellular end of IL-2Rβ/*γ*_c_. Activation of the JAK/STAT pathway can further initiate the downstream PI3K/AKT/mTOR, Ras/Raf/MAPK, and AKT-XBP1s pathways, which together induce the expression and activation of c-myc, c-fos, c-jun, Bcl-2, Bcl-xL, and NF-κB, and reduce the expression of Bim and PUMA, ultimately promoting the survival, proliferation, and activation of effector cells (Figure 2) (Waldmann, 2015; Guo et al., 2017; Zhang et al., 2021).

**FIGURE 3:**
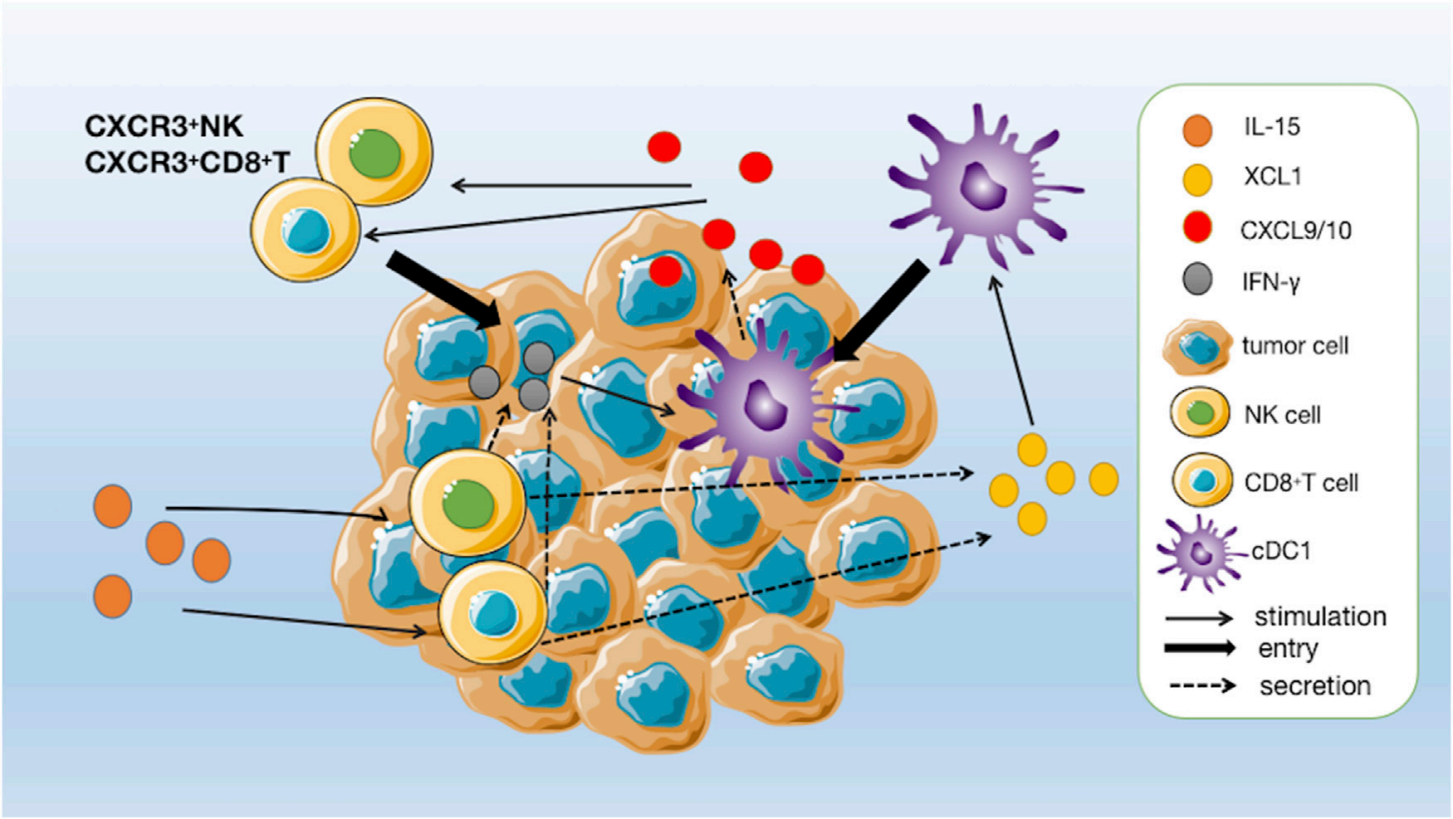
A positive immune feedback loop for IL-15. CD8^+^ T cells and NK cells are activated by IL-15 and release XCL1. The increased concentrations of XCL1 recruit conventional type I dendritic cells (cDC1) to pre-reside in tumour tissue. CD8^+^ T cells are induced to activate by cDC1 and enter the tumour to kill tumour cells and, together with activated NK cells, secrete IFN-γ to further stimulate cDC1 to secrete chemokines CXCL9 and CXCL10, thereby increasing the number of circulating CXCR3^+^ NK and CD8^+^ T cells, which can further infiltrate the tumour following a gradient of CXCL9 and CXCL10 chemokines.

Among the many immune cells that express IL-2Rβ/*γ*_c_, NK cells, and CD8^+^ T cells are particularly responsive to IL-15 stimulation (Zhang et al., 2021). One of the primary mechanisms by which IL-15 exerts its immunostimulatory effects is the activation and expansion of NK cells. The upregulation of Bcl-2 and Ki67 by IL-15 stimulation promotes the survival and proliferation of NK cells and CD8^+^ T cells while upregulating the transcription of genes such as Gzmb, Gzma, Prf1, and CD69, and upregulating the secretion of perforin, granzyme B and INF-γ, which manifests as enhanced cell killing activity (Bergamaschi et al., 2020; Bergamaschi et al., 2021; Zhang et al., 2021). Di Pilato and Nolz and Richer, (2020) found that IL-15 plays a crucial role in the homeostatic survival and proliferative activation of memory T cells and the maintenance of effector-like cytotoxic T lymphocytes (CTLs) in the tumor microenvironment Di Pilato et al., 2021). In addition, IL-15 has been found to enhance the antibody-dependent cell-mediated cytotoxic effects on NK cells and the efficacy of conjugated therapeutic anti-tumor monoclonal antibodies (Zhang et al., 2018). Recent studies have found that tumor-infiltrating NK cells and CD8^+^ T cells activated by IL-15 upregulate the secretion of a chemokine named XCL1 (Bergamaschi et al., 2020). XCL1 can recruit conventional type I dendritic cells (cDC1) to colonize the tumor tissue. Moreover, cDC1 has a strong ability to cross-presenting antigen, which can effectively induce the activation of initial CD8^+^ T cells in tumor-draining lymph nodes to enter the tumor foci to synergistically kill tumor cells, and further enhance the anti-tumor effect (Bergamaschi et al., 2020). In addition, IFN-γ secreted by activated NK cells and CD8^+^ T cells stimulates cDC1 to secrete CXCL9 and CXCL10, which can recruit peripheral CXCR3^+^ NK and CD8^+^ T cells to tumor tissue, forming an immune positive feedback loop that maintains immune surveillance against tumors (Figure 3) (Bergamaschi et al., 2020).

IL-15 has also been investigated as a potential therapeutic agent for the treatment of various types of cancer. For example, upregulation of IL-15 improves cancer-testis antigens (CTA) invasion. This tumor antigen screening strategy that could be used to develop therapeutic vaccines against non microsatellite instability-high (non-MSI-H) colorectal cancer (CRC) (Shi et al., 2023). Moreover, Tumino et al. (2023) proposed that IL15 could be used as a novel strategy to increase the efficacy of NK-based immunotherapy in a variety of currently refractory primary and metastatic cancers of solid tumors in adults and children. In addition, activation of IL-15 can be used as a promising therapeutic strategy for pancreatic ductal adenocarcinoma (PDA) (Kurz et al., 2022) and IL-15 is also one of the most promising prognostic markers for patients with biliary tract cancer (BTC) (Christensen et al., 2023). And the same results can be found in multiple mouse models: primary and metastatic tumors can be inhibited by IL-15. For instance, transducing the colon carcinoma cell line MC-38 with IL-15Rα in the mouse colon cancer model inhibits MC-38 pulmonary metastases *in vivo* in mice (Zhang et al., 2009). In the mouse metastatic breast cancer model, high amount of metastases in IL-15^−/−^ mice but near-abolition of tumor metastases in IL-15Tg and IL-15-treated mice compared with wild-type (WT) mice (Gillgrass et al., 2014). Furthermore, Nandi et al. (2023) reported that IL-15 signaling in the thymus controls T-acute lymphoblastic leukemia (T-ALL) development and that NOD.Scid mice lacking interleukin-15 (IL-15) or IL-15Rα may develop T-ALL. In conclusion, IL-15 provides an exciting opportunity to enhance cancer immunotherapy.

## 5 Progress in the development of IL-15 agonists

IL-15 is a promising immunotherapeutic agent owing to its powerful immune-activating effects. Therefore, IL-15 derivatives have garnered attention as potential immunotherapeutic agents for cancer. Several promising IL-15 agonists have been uncovered through preclinical studies, but only a small number have successfully entered clinical trials. More than 170 clinical trials using different IL-15 agents for cancer treatment have been initiated worldwide, but no IL-15 agonist drug/therapy has been approved for marketing so far (Waldmann et al., 2020a; Waldmann et al., 2020b). Below we summarize the history of IL-15 agonist development and present the representative molecules and related findings.

### 5.1 Recombinant human interleukin-15

IL-15 agonists have been developed in three main phases. In the initial phase, researchers developed the first IL-15 agonist, recombinant human interleukin-15 (rhIL-15), through recombinant technology. rhIL-15 is an approximately 13 kDa, non-glycosylated IL-15 monomer produced in *E coli* (Conlon et al., 2015). In 2015, Conlon *et al.* published the results of the first-in-human phase I clinical trial of rhIL-15, which was conducted on patients with metastatic malignant melanoma or metastatic renal cell carcinoma. rhIL-15 was administered by intravenous bolus (IVB) infusion over 12 consecutive days, and the results showed that rhIL-15 at the maximum tolerated dose increased the number of peripheral NK cells and CD8^+^ T cells to some extent. However, no objective remission was observed in any of the 18 patients, with the best outcome being a stable disease, while dose-dependent toxicities such as fever and chills, rigors, thrombocytopenia, and hypotension were also observed. And the mean plasma half-life was approximately 2.5 h following intravenous bolus administration (Conlon et al., 2015). In another study, a transient drop in circulating neutrophils was observed at higher doses of IL-15, along with accumulation of granulocytes in the liver (Waldmann et al., 2011). The team further conducted a first-in-human outpatient phase I dose escalation trial of subcutaneous (SC) rhIL15 in refractory solid tumor cancer patient, and the results showed that SC rhIL15 treatment resulted in significant increases in circulating NK and CD8^+^ T cells, and the treatment was well tolerated: only 2 of 19 patients had serve adverse events (SAE) (grade 2 pancreatic and grade 3 cardiogenic chest pain with hypotension and elevated troponin) (Miller et al., 2018). In 2019, Conlon et al. (2019) continued a phase I trial of rhIL15 administered as a 10-day continuous intravenous infusion (CIV) to patients with cancers. This administration could produce significant expansions of CD8^+^ T and NK effector cells in circulation and tumor deposits, but no significant improvement was observed. At present, a new administration method of rhIL-15 has been attempted in the latest research. Rebhun et al. (2022) conducted a Phase I trial of inhaled rhIL-15 in 21 dogs with gross pulmonary metastases and established 50 µg twice daily × 14 days as the maximum tolerated dose (MTD) and recommended phase 2 dose. They observed promising clinical activity with an overall clinical benefit of 39% when administered as a monotherapy for only 14 days. Even so, the results of these trials of rhIL-15 showed its shortcomings, including short half-life (requiring continuous daily dosing). Therefore, the optimal dose and schedule of administration of recombinant human IL-15 for different diseases remain to be determined, and further studies are needed to fully understand its potential as a therapeutic agent. In conclusion, recombinant human IL-15 holds promise as a potential treatment for cancer and viral infections. However, clinical trials must carefully evaluate its safety and efficacy to determine its optimal use in various diseases.

### 5.2 IL-15 modification and IL-15/IL-15Rα complex

To overcome the shortcomings of rhIL-15, two main optimization ideas arose in subsequent studies: one is to introduce mutations or modifications to IL-15 itself, which is a common modification strategy for protein drugs, and the other is the development of IL-15/IL-15Rα complexes. Since IL-15 acts as hetIL-15 *in vivo*, researchers hypothesized that IL-15/IL-15Rα complexes have better activity and stability as a drug molecule compared to IL-15 monomers, which can simulate the *in vivo* action of IL-15. This hypothesis is confirmed in subsequent studies (Van den Bergh et al., 2017; Bergamaschi et al., 2021). The development of IL-15 agonists has thus entered a second phase. Several IL-15 agonist molecules have been successfully developed and have been clinically studied, such as SO-C101 (SOT-101) (Desbois et al., 2020), hetIL-15 (NIZ985) (Conlon et al., 2021), P22339 (SHR-1501) (Hu et al., 2018), NKTR-255 (Miyazaki et al., 2021), HCW9218 (Liu et al., 2021), HCW9201 (Romee et al., 2012), ALT-803 (N-803) (Wong et al., 2013), BJ-001, OBX-115, etc.

SO-C101 (SOT-101), a potent transdermally administered IL-15Rβγ super agonist developed by Mortier et al., is a human fusion protein formed by linking IL-15 to the NH(2) terminal (amino acids 1–77, sushi^+^) domain of IL-15Rα(Mortier et al., 2006), which prolongs the half-life of IL-15 and promotes the development and differentiation of NK cell (Huntington et al., 2009). Desbois et al. (2016) has shown that SO-C101 enhances the anti-tumor activity of anti-PD-1 agents in a colorectal cancer mouse model by promoting the proliferation and function of CD8^+^ T cells *in vivo*. SO-C101 can also kill tumor cells and reduce metastatic disease by increasing NK cell infiltration, maturation, and proliferation while reducing the number of lung-infiltrating neutrophils (CD11b^+^Ly6G^high^Ly6C^low^) (Desbois et al., 2020). On 8 December 2021, SOTIO Biotech decided to conduct a Phase 2 clinical trial on the efficacy and safety of SO-C101 in combination with the PD-1 inhibitor Keytruda (pembrolizumab) in patients with advanced/refractory solid tumors, which has yielded clinical benefits and an encouraging safety profile (Table 2).

**TABLE 2:** Examples of ongoing clinical trials with IL-15.

Drug	Other name	Status	Quantity	*NCT number*	Responsible party	Conditions or diease	Combination drug	Phase	*Start Date*	Completion date
SO-C101	SCT101	Recruiting	2	NCT04234113	SOTIO Biotech (SOTIO Biotech AG)	Selected advanced/metastatic solid tumo	Alone/combine with pembrolizumab	1/1b	2019.6.13	2023.12
				NCT05256381		Selected advanced/reefractory solid tumors	Combine with pembrolizumab	2	2022.6.21	2023.9.22
0BX-115	—	Recruiting	1	NCT05470283	M.D. Anderson cancer center	Metastatic melanoma	Combine with acetazolamide	1	2022.9.7	2027.4.1
NKTR-255	—	Recruiting	4	NCT04616196	Nektar Therapeutics	Ead and neck squamous cell carcinoma, colorectal carcinoma, cutaneous squamous cell carcinoma	Alone/combine with cetuximab	1b/2	2020.10.30	2023.6
				NCT05359211	Fred Hutchinson Cancer Center	Relapsed or refractory large B-cell lymphoma	Combine with CAR-T cell therapy	1b/2	2022.11.18	2024.1.31
				NCT03233854	Crystal Mackall, MD, Stanford University	CD19-positive B acute lymphoblastic leukemia who had relapsed or had no response to therapy	Combine with CD19/CD22 CART cells and chemotherapy	1	2017.9.1	2025.9.1
				NCT05327530	EMD Serono	Locally Advanced or Metastatic Urothelial Carcinoma	Combine with avelumab	2	2022.8.17	2026.8.5
		Enrolling by invitation	1	NCT04136756	Nektar therapeutics	Multiple myeloma non-hodgkin lymphomaIndolent non-hodgkin lymphoma	Alone/combine with Rituximab or daratumumab	1	2019.10.7	2023.3
BJ-001	—	Recruiting	1	NCT04294576	BJ Bioscience, Inc	Locally advanced/metastatic solid tumors	Alone/combine with pembrolizumab	1	2019.12.4	2024.4.29
NIZ985	IL-15/sIL-15Ra, hetIL-15	Recruiting	1	NCT04261439	Novartis Pharmaceuticals	Relapsed advanced solid tumors and lymphoma	Alone/combine with spartalizumab/islelizumab	I/Ib	2020.2.27	2024.3.11
		Completed	1	NCT02452268		Metastatic and advanced solid tumors	Alone/combine with PDR001	I/Ib	2017.5.8	2022.3.7
HCW9218	Monotherapy	Recruiting	2	NCT05322408	Masonic cancer center, niversity of minnesota	Advanced/metastatic solid tumor cancer (Except pancreatic and primary brain cancers)	Alone	1	2022.4.1	2027.1
				NCT05304936	HCW Biologics	Advanced pancreatic carcinoma		1b/2	2022.10.17	2023.3.1
SHR-1501	P22339	Active, not recruiting	1	NCT03995472	Jiangsu HengRui Medicine Co., Ltd	Advanced malignancies	Combine with SHR-1316	1	2020.2.14	2020.9
		Not yet recruiting	1	NCT05410730	Shanghai hengrui pharmaceutical Co., Ltd	Non-muscle invasive bladder cancer	Alone/combine with BCG	1/2	2022.6	2025.5

Data are referenced from https://clinicaltrials.gov/.

HetIL-15 (NIZ985) is a recombinant isomer of physiologically active interleukin-15 and IL-15Rα prepared by Conlon et al. (2021). The molecule is highly homologous to naturally occurring IL-15 circulating in human plasma (Chertova et al., 2013). It has shown superior pharmacokinetics (PK) in several studies in mice and macaques. It promotes cytotoxic lymphocyte proliferation, killing function, and organ/tumor infiltration, resulting in anti-tumor effects (Chertova et al., 2013). In the B16 melanoma, MC38 colon carcinoma, TC-1 carcinoma, breast, and pancreatic tumor mouse models, hetIL-15 therapy was effective against both primary tumor and metastatic disease (Ng et al., 2017; Bergamaschi et al., 2020). In 2020, Leidner et al. (2020) reported the interim data from the first human study of NIZ985, which evaluated the safety of subcutaneous NIZ985 as a single drug (SA) or in combination (CM) dosing with the PD-1 inhibitor spartazizumab in adults with metastatic/unresectable solid tumors. The results showed that NIZ985 displays approximately dose-proportional, time-dependent pharmacokinetics and that its antitumor activity was limited during dose escalation. However, preliminary responses in both immuno-oncology treatment (IO)-experienced and IO-naïve patients were seen in combination with spartalizumab (Leidner et al., 2020). And the first human dose-increasing study with NIZ985 as a single drug evaluated the safety and preliminary activity of NIZ985. The patients with advanced cancer received a subcutaneous injection of NIZ985 three times a week in this study and the results confirmed that the tolerance was good. The immune activation produced by NIZ985 is similar to that observed preclinically and can induce IFN- *γ* And cytotoxic lymphocyte proliferation (Conlon et al., 2021). The latest findings of demonstrate a significant anti-metastatic potential of hetIL-15 in combination with chemotherapy and surgery and suggest exploring the use of this regimen for the treatment of metastatic triple negative breast cancer (TNBC) (Stravokefalou et al., 2022). In addition, another clinical trial showed that treatment with hetIL-15 could also inhibit tumor growth, prolong survival time and enhances the sensitivity to chemotherapy of pancreatic ductal adenocarcinoma (PDA) (Kurz et al., 2022). HetIL-15 was initially synthesized by Dr. George Pavlakis’s group (NCI-Frederick) licensed initially to Admune Therapeutics that produced the clinical grade material used in the FIH clinical trial. Later the agent (and intellectual property rights) was wholly purchased by Novartis, and the clinical trials continue under the name NIZ985. Its highest development status is in clinical phase 1 (Table 2).

SHR-1501 is an interleukin-15 (IL-15) fusion protein developed by Hengrui Pharmaceuticals with intellectual property rights, which constructs a complex of IL-15 and the sushi structural domain of the IL-15Rα chain. SHR-1501 enhances IL-15 agonist activity through transfection and creates a disulfide bond linking the IL-15/Sushi structural domain complex to IgG1-Fc, thus extending its half-life (Hu et al., 2018). SHR-1501 stimulates the proliferation of T cells, B cells, and NK cells *in vivo*, and mobilizes the body’s immune system to remove foreign substances such as tumors. In contrast to IL-2, IL-15 does not stimulate Treg proliferation and induces T-cell apoptosis (Hu et al., 2018). In March 2022, SHR-1501 for injection was approved for a phase I/II clinical study of bladder perfusion to treat non-muscle invasive bladder cancer (NMIBC) in both dose-escalation and dose-expansion. To date, two Phase I clinical trials of SHR-1501 for injection in advanced tumors have been conducted in China and Australia (Table 2).

NKTR-255 is a novel rhIL-15 polyethylene glycol coupling designed by Miyazaki et al. (2021) that retains the ability to bind to all receptors and maintains the full biological properties of IL-15 . Compared to rhIL-15, NKTR-255 has better PK properties and a substantially longer half-life (15.2 h vs. 0.168 h). At the same time, NKTR-255 induces sustained receptor engagement, efficiently and durably stimulates proliferation and activation of NK cells and CD8^+^ T cells, and shows superior anti-tumor activity to rhIL-15 (Miyazaki et al., 2021). These results support the advancement of NKTR-255 to the clinic, with its first phase 1 clinical trial now completed in patients with relapsed or refractory multiple myeloma and non-Hodgkin’s lymphoma (Shah et al., 2021). In addition, data from Fernandez et al. showed the same results: NKTR-255 had important effects and translational significance in inducing NK cells to function against multiple myeloma cells (Fernandez et al., 2023). Currently under investigation by Nektar Therapeutics and EMD Serono, the research on metastatic urothelial cancer has been in clinical phase 2 (Table 2).

In addition, HCW Biologics has developed HCW9218, a bifunctive molecule containing IL-15 agonist functions, based on the TOB™ platform. It's a heterodimeric fusion protein containing soluble TGF-βRII and IL-15/IL15RαSu, which can neutralize TGF-β (inhibiting tumor progression and relieving immunosuppression) and directly activate immune cells. It is an excellent example of the rational design of a bifunctional molecular that can effectively inhibit tumor growth, prolong survival, and has a good safety profile when used alone, and its clinical trials on patients with advanced pancreatic cancer are in phase 1b/2 (Table 2) (Liu et al., 2021). In 2022, the research found that HCW9218 could make chemotherapy, therapeutic antibodies, and checkpoint blockade more effective by killing therapy induced senescence (TIS) cancer cells while reducing TIS-mediated proinflammatory side effects in normal tissues (Chaturvedi et al., 2022). The latest study found similar results, saying HCW9218 could be safely administered systemically to reduce senescent cells and alleviate senescence-associated secretory phenotype in mice. It indicates that HCW9218 represents a novel immunotherapy and a clinically promising new class of senotherapeutic agents targeting cellular senescence-associated diseases (Shrestha et al., 2023).

HCW9201 is also an IL-15 agonist developed by HCW Biologics based on its TOB™ platform. It is a fusion protein containing IL-12, IL-15, and IL-18 receptors, which can activate and amplifies memory-like (ML) NK cells, prolong life and enhances anti-cancer function *in vitro* (Romee et al., 2012; Tarannum and Romee, 2021). HCW9201 is currently mainly used in the treatment of leukemia. In 2021, Becker-Hapak et al. (2021) found that HCW9201 showed similar increases in short-term and memory-like NK cell cytotoxicity and IFN production against leukemia targets, with equivalent control of leukemia in NSG mice. In the same year, two clinical trials both confirmed that combined treatment with ML NK cells could produce good therapeutic effects on leukemia. For example, Bednarski et al. (2022) confirmed that the combination of ML NK cells and donor lymphocyte infusions could provide a new immunotherapy platform for relapsed acute myeloid leukemia after allogeneic hematopoietic cell transplantation. Berrien-Elliott et al. (2022) confirmed that ML NK cell therapy combined with N-803 support safely enhanced the treatment of acute myeloid leukemia with reduced-intensity conditioning haplotype hematopoietic cell transplantation.

ALT-803 is a 2 (IL-15/IL-15Rα) tetramer formed by the assembly of two IL-15 mutants (IL-15 N72D, which has a higher affinity for the receptor) and two IL15Rα Su-IgG1 Fc fusion proteins (Wong et al., 2013). It was renamed as N-803 after being purchased by ImmunoBio. The safety, pharmacokinetics, and immune effects of N-803 have been studied in mice and crab monkeys. Treatment of mice with N-803 showed a 25 h half-life and a 5–20-fold increase of *in vivo* bioactivity (Han et al., 2011). And in crab monkeys, N-803 induced dose-dependent increases of NK, CD4^+^, and CD8^+^ memory T cells. Pharmacokinetic analysis revealed that the half-life was approximately 7.2–8 h. Phase I clinical trials have been completed with N-803 as a single agent, and in combination with nivolumab in a trial of N-803 monotherapy for hematologic malignancies, 19% of 33 patients showed remission, with three patients in stable disease, one in partial remission, and one in complete remission (Romee et al., 2018). In a trial of N-803 in combination with nivolumab in patients with metastatic non-small cell lung cancer, 10 of 21 patients had stable disease, and six were in partial remission. The results demonstrated good tolerability of N-803 treatment, the ability to use a once-weekly dosing strategy, and preliminary validation of its efficacy. But as the experiment progressed, the researchers found a problem: Seven (33%) of the 21 patients in the study developed detectable anti-ALT-803 antibody titres in sera after multidose treatment and 3 of the 7 (42%) responded to treatment (all partial responses), which may be the reason why the immune response in the second cycle of treatment was significantly lower than that in the first cycle (Wrangle et al., 2018). The same problem was found in another clinical trial in which mike et al. administered ALT-803 intravenously or subcutaneously to patients with incurable advanced melanoma, renal cells, non-small cell lung cancer, and head and neck cancer. Although patients did not develop drug-resistant antibodies, the total number of circulating CD4^+^ and CD8^+^ T cells (including CD8^+^ memory T cells) did not increase significantly after sequential treatment with ALT-803, and in six of the eight subcutaneously treated patients who continued treatment after the first cycle, the total number of circulating NK cells did not increase beyond the level of cycle 1 (Margolin et al., 2018). Therefore, these results suggested that prolonged continuous treatment might reduce biological responsiveness to subsequent treatment. Additional trials of N-803 in combination with other monoclonal antibodies are underway (Bergamaschi et al., 2021), with the principal investigators currently being ImmunityBio, Inc. and the National Institutes of Health Clinical Center. Stage 3 or 4 advanced or metastatic non-small cell lung cancer is currently in clinical Phase 3 development. (Table 3).

**TABLE 3:** Examples of ongoing clinical trials with N-803.

Staus	Quantity	*NCT number*	Responsible party	Conditions or diease	Combination drug	Phase	Start Date	Completion date
Recruiting	9	NCT03022825	Immunity Bio, Inc	BCG unresponsive high grade non-muscle invasive bladder cancer (NMIBC)	Alone/combine with BCG	II/III	2017.6.2	2023.1
		NCT02138734		Non-Muscle Invasive Bladder Cancer	Combine with BCG	Ib/IIb	2014.7.21	2025.12
		NCT04898543		Newly diagnosed high-risk solid tumors who have not received prior treatment for high-risk tumors or relapsed/refractory (r/r) solid tumors who have progressive disease after receiving ≥ 2 prior therapies	N-803/M-CENK	1	2021.6.21	2022.6
		NCT04390399		Locally advanced or metastatic pancreatic cancer	Combine with standard-of-care chemotherapy, aldoxorubicin HCl, and PD-L1 t-haNK	2	2020.7.21	2024.6.30
		NCT04491955	National Institutes of Health Clinical Center	Metastatic or refractory/recurrent small bowel and colorectal cancers	Combine with MVA-BN-CV301 and M7824	2	2020.9.22	2024.7.31
		NCT03493945		Metastatic prostate cancer and solid tumor	Combine with BN-brachyury, M7824, and Epacadostat	I/II	2018.5.1	2023.12.31
		NCT04847466		Advanced gastric or head and neck cancer who have already had standard cancer treatment	Combine with pembrolizumab and PD-L1 t-haNK	2	2021.12.14	2025.1.31
		NCT05096663	Southwest Oncology Group	Stage IV or recurrent non-small cell lung cancer previously treated with anti-PD-1 or anti-PD-L1 therapy	Combine with pembrolizumab	II/III	2022.2.15	2027.2.1
		NCT04290546	Glenn J. Hanna, Dana-Farber Cancer Institute	Squamous cell carcinoma of the head and neck recurrent head and neck squamous cell carcinoma	Combine with CIML NK cell and pilimumab	I	2020.7.20	2022.12.31
Not yet recruiting	2	NCT05445882	National Institutes of Health Clinical Center	Castration resistant prostate cancer	Combine with bintrafusp alfa/BN-brachyury/bintrafusp alfa + BN-brachyury	II	2022.11.10	2023.11.1
		NCT05419011	National Cancer Institute	Lynch syndrome	Combine with TRI-AD5	IIB	2022.11.1	2026.10.1
Active, not recruiting	6	NCT04247282	National Institutes of Health Clinical Center	Head and neck cancer	Combine with M7824 and TriAd vaccine	I/II	2020.6.9	2023.6.1
		NCT04927884	ImmunityBio, Inc	Triple negative breast cancer (TNBC) after at least 2 prior treatments for metastatic disease	Combine with PD-L1 t-haNK, sacituzumab govitecan-hziy and drug: cyclophosphamide	Ib/II	2021.9.27	2024.1
		NCT04927884		Stage 3 or 4 advanced or metastatic non-small cell lung cancer	Combine with the current standard of care	III	2018.5.18	2023.7.1
		NCT03387085		NANT triple negative breast cancer	Combine with avelumab and bevacizumab, etc.	Ib/2	2018.3.16	2022.12
		NCT03228667		Non-small cell lung cancer, small cell lung cancer, urothelial carcinoma, etc.	Cmobine with pembrolizumab/nivolumab/atezolizumab, etc.	IIb	2018.12.1	2023.5
		NCT02989844	Masonic Cancer Center, University of Minnesota	Acute myelogenous leukemia and myelodysplastic syndrome following reduced intensity conditioning allogeneic stem cell transplantation	Alone	II	2017.4.12	2022.1.10

Data are referenced from https://clinicaltrials.gov/.

Recent years have seen a golden era in the development of IL-15 agonists, with many innovative designs being proposed and several new targeted/multifunctional IL-15 agonists emerging. These include several specific and promising molecules, such as pro-IL-15, the first IL-15 agonist molecule to use a prodrug design strategy. Jingya Guo’s team used IL-15-IL15RαSu-Fc as the basis and attached an extracellular structural domain of IL-15Rβ to form pro-IL-15 via an MMP-14 cleavable peptide linker at the N-terminal end. Pro-IL-15 blocks the binding site of IL-15 and renders it inactive in the peripheral circulation. In the matrix metalloproteinase-rich (MMP) tumor microenvironment, the peptide linker is enzymatically cleaved to allow IL-15Rβ to be shed, thereby restoring the molecule’s activity. The results showed that pro-IL-15 activated CD8^+^ T cells in the tumor reduced systemic toxicity and inhibited tumor growth (Guo et al., 2021). There is also a very clever and novel molecule, anti-mPD1-IL15m, which consists of a fusion of a PD-1 specific antibody and a “low affinity” IL-15 mutant. Anti-mPD1-IL15m precisely activates PD1^+^ TILs in tumors with little effect on peripheral NK cells and T cells. Anti-mPD1-IL15m exhibits more substantial anti-tumor effects than IL-15 super agonists/anti-mPD1/both combinations and is a promising molecule with a better safety profile than IL-15 super agonists (Xu et al., 2021).

## 6 Combination of IL-15 with adoptive cell therapy

In addition to using IL-15 and its agonists alone in cancer immunotherapy, IL-15 has been incorporated into many cancer-specific pericyte therapies, particularly in combination with chimeric antigen receptor (CAR) engineering. Chimeric antigen receptor engineered T (CAR-T) or Chimeric antigen receptor engineered NK (CAR-NK) cells are considered adoptive cell therapy and represent a promising anticancer strategy.

In 2017, the United States Food and Drug Administration approved the first anti-CD19 CAR-T cell therapy for hematologic malignancies (June et al., 2018). CAR-T cells amplified *ex vivo* with IL-15 were characterized by a less differentiated phenotype, decreased expression of exhaustion and pro-apoptotic molecules, improved mitochondrial metabolism, and infusion demonstrated a greater anti-tumor response *in vivo* (Singh et al., 2016; Alizadeh et al., 2019). In Hoyos et al., researchers engineered T (iC9/CAR.19/IL-15 T) cells with a retroviral vector encoding anti-CD19 CAR, IL-15, and inducible caspase-9-based suicide gene (iC9). The IL-15-enhanced CAR T cells were able to expand 10-fold *in vitro* and 3- to 15-fold *in vivo*, and in the SCID lymphoma human xenograft model, the iC9/CAR.19/IL-15 T cells demonstrated greater effectiveness, along with better persistence and anti-tumor effects (Hoyos et al., 2010). And in another human CD19 CAR-T study, high IL-15 plasma levels at CAR-T infusion were associated with the efficacy of this therapy (Kochenderfer et al., 2017). A sequence encoding for a membrane-bound IL-15 (mbIL-15) fusion protein has been incorporated into the CAR-expressing lentiviral vectors, leading to the development of a new generation of CAR-T cell-based therapies. CD19 CAR-T cells co-expressing mbIL-15 have long life and can not only enhance NK cell activation and reduce the number of M2 macrophages, but also play a good anti-tumor role in the leukemia mouse model and the glioblastoma mouse model (Hurton et al., 2016; Krenciute et al., 2017). Morever, the latest research claimed that the CAR T cells modified by IL-15 can be used as double targeting drugs for tumor cells and myeloid-derived suppressor cells in glioblastoma (Zannikou et al., 2023).

Another popular immune cell population for cancer immunotherapy is NK cells. When IL-15 or IL-15 receptor complex is overexpressed in NK cells, the survival rate of NK cells can be significantly increased and the antitumor effect is enhanced (Zhou et al., 2022). In a Phase I/II clinical trial, researchers used cord blood (CB)-derived iC9/CAR.19/IL15 CB-NK cells to treat refractory B-cell lymphoma or leukemia. Of the 11 patients involved in the study, 73% responded at all dose levels within 30 days after infusion, with infused CAR NK cells lasting at least 12 months (Liu et al., 2020). However, studies have found severe toxicity, even fatal, in IL-15-regulated NK cell therapy, possibly due to high levels of circulating human pro-inflammatory cytokines accompanied by a dramatic expansion of NK cells causing severe inflammation or cytokine release syndrome (Christodoulou et al., 2021a; Christodoulou et al., 2021b).

## 7 Summary and discussion

IL-15 has shown significant potential in cancer immunotherapy by enhancing the anti-tumor activity of immune cells and promoting the development of memory T cells. Recent research has focused on optimizing IL-15 delivery methods and combining it with other immune modulators to increase its efficacy and reduce potential side effects. Preclinical and clinical studies have demonstrated the efficacy and safety of IL-15 in various tumor models and cancer patients, respectively. Efficacy, safety, and longevity are the focus of IL-15 agonist development and optimization, and striking a balance among the three is the key to success. In terms of improving safety, local activation of the immune response and reduction of systemic toxicity is currently achieved by targeting IL-15 agonists to the tumor microenvironment or specific immune cells among them (Xu et al., 2021). On this basis, as the enrichment of IL-15 agonists to tumors disguisedly reduces the affinity requirement of IL-15 to the receptor, one study has provided an idea its action at peripheral non-target sites can be further reduced and safety improved by appropriately reducing its receptor affinity (Xu et al., 2021). In terms of improving efficacy, improving tumor targeting is also a strategy to improve effectiveness. Non-targeted IL-15 agonists mainly act on immune cells in the periphery, lymph nodes, and spleen. Their anti-tumor effects depend on their ability to enter the tumor foci and maintain activation, which may explain the suboptimal efficacy of some non-targeted IL-15 agonists in solid tumors (Margolin et al., 2018; Miller et al., 2018).

In contrast, tumor-targeted IL-15 agonists mainly act on immune cells within the tumor, allowing them to exert anti-tumor effects more consistently. Theoretically, the whole mechanism of action becomes more direct. To some extent, it can overcome the problem of difficult penetration of immune cells into the tumor and thus should exert better anti-tumor effects (Corbellari et al., 2021; Xu et al., 2021). On the other hand, IL-15 agonists can also achieve increased effectiveness by combining with suitable drug molecules (e.g., immune checkpoint inhibitors, therapeutic anti-tumor monomers, immunomodulatory molecules, etc.) or by constructing multifunctional molecules to produce synergistic effects. Therefore, finding the right combination may require more in-depth studies of the mechanisms in the future (Waldmann et al., 2020a; Waldmann et al., 2020b). Longevity is also the current focus of optimization of IL-15 agonists, and PEG modification, protein fragment fusion, and construction of particulate formulations are commonly used to enable reduced clearance, increased retention, and slow release to achieve extended half-life and improved PK properties (Wong et al., 2013; Liu et al., 2021; Miyazaki et al., 2021; Hangasky et al., 2022). In summary, the rational design of IL-15 agonists is the focus of future research and development. Researchers need to consider about how to meet the three needs mentioned above through limited modification design. In addition, besides using of traditional recombination and modification techniques, the combination of IL-15 and adoptive cell transfer has shown remarkable outcomes in treating B-cell lymphoma, AML, neuroblastoma, and glioblastoma. Despite its potent antitumor effect, the dramatic NK cell expansion it caused potentially led to the lethal death of treated mice, raising concerns about the safety of IL-15 armored immune cells in cell therapy (Zhou et al., 2022).

In conclusion, despite the promising results of IL-15 in preclinical and clinical studies, several challenges remain in the clinical translation of IL-15 therapy. Therefore, further research is needed to optimize IL-15 delivery and dosing regimens to improve its efficacy and reduce side effects.
